# Optimal timing of lacrimal duct probing for congenital nasolacrimal duct obstruction in infants: A retrospective cohort study

**DOI:** 10.1097/MD.0000000000049230

**Published:** 2026-06-12

**Authors:** Xiaolan Xie, Shana Wang, Xi Qin, Jingjing Wang, Bidan Zhu

**Affiliations:** a Department of Ophthalmology, Tongzhou Maternal and Child Health Hospital of Beijing, Beijing, China.

**Keywords:** congenital nasolacrimal duct obstruction, lacrimal duct probing, retrospective cohort study, surgical timing

## Abstract

The optimal timing of lacrimal duct probing for congenital nasolacrimal duct obstruction (CNLDO) remains debated. This study aimed to evaluate the association between age at probing and short term treatment outcomes in infants with CNLDO. This retrospective cohort study analyzed 394 affected eyes from 304 infants aged 3 to 24 months who were diagnosed with CNLDO and underwent lacrimal duct probing at the Ophthalmology Clinic of Tongzhou Maternal and Child Health Hospital of Beijing between August 2019 and July 2024. Patients were categorized into 3 groups according to age at probing: Group A, 3 to 6 months; Group B, 7 to 12 months; and Group C, 13 to 24 months. Short term clinical resolution rates were compared among the groups. Overall, 386 of 394 eyes achieved clinical resolution, corresponding to an overall success rate of 98.0% (95% confidence interval [CI]: 96.0–99.1%). The success rate after the first probing was 87.3% (95% CI: 83.6–90.4%), and the success rate after the second probing was 84.0% (95% CI: 70.9–92.8%). The total success rates were 99.6% in Group A, 97.8% in Group B, and 86.7% in Group C. A significant difference was observed among the 3 groups (*P* < .001). The success rates in Groups A and B were significantly higher than that in Group C, whereas no significant difference was observed between Groups A and B. Lacrimal duct probing was associated with a high short term success rate in infants with CNLDO, particularly when performed within the first 12 months of life. Because of the retrospective design, limited follow up duration, and small number of patients older than 12 months, these findings should be interpreted as supportive evidence for timely probing rather than definitive proof of an optimal surgical age.

## 1. Introduction

Congenital nasolacrimal duct obstruction (CNLDO) is one of the most common ocular disorders in pediatric ophthalmology, with a reported incidence ranging from 5% to 20% among neonates worldwide.^[1–3]^ It is most commonly caused by incomplete canalization or persistence of a membranous obstruction at the distal end of the nasolacrimal duct, often at the valve of Hasner, resulting in impaired tear drainage. Clinically, CNLDO may present with persistent or intermittent epiphora, mucopurulent discharge, recurrent conjunctivitis, and reflux of mucous or purulent material from the lacrimal punctum after pressure over the lacrimal sac.

Initial management of CNLDO is usually conservative because many cases resolve spontaneously during infancy. Conservative treatment commonly includes lacrimal sac massage and topical antibiotics when mucopurulent discharge or conjunctival inflammation is present.^[4–6]^ However, surgical intervention may be required when symptoms persist despite conservative management. Lacrimal duct probing remains a widely used first-line surgical procedure because it is simple, minimally invasive, and generally associated with high success rates.

The optimal timing of lacrimal duct probing remains debated. Some clinicians favor continued conservative management during the first year of life, particularly in children with simple membranous obstruction, and reserve probing for persistent cases. In contrast, other studies have reported reduced probing success with increasing age, possibly related to chronic inflammation, fibrosis, or more complex obstruction.^[7–11]^ Therefore, further clinical data are needed to clarify the relationship between age at probing and treatment outcomes.

In this retrospective cohort study, we evaluated infants with CNLDO who underwent lacrimal duct probing at a single maternal and child health hospital between August 2019 and July 2024. By comparing short-term clinical resolution rates among different age groups, we aimed to assess the association between age at probing and treatment outcome, and to provide practical evidence to support clinical decision-making regarding timely intervention.

## 2. Patients and methods

### 2.1. Study design and patients

This retrospective cohort study was conducted at the Department of Ophthalmology, Tongzhou Maternal and Child Health Hospital of Beijing, China. Clinical data were collected from pediatric patients diagnosed with CNLDO who underwent lacrimal duct probing between August 2019 and July 2024.

A total of 304 infants involving 394 affected eyes were included in the analysis. The cohort included 165 males and 139 females. Bilateral involvement was present in 90 patients, while 214 patients had unilateral disease, involving 199 right eyes and 195 left eyes.

To evaluate the association between age at probing and treatment outcome, patients were divided into 3 groups according to age at the time of probing: Group A, 3 to 6 months; Group B, 7 to 12 months; and Group C, 13 to 24 months. Group A included 174 patients and 229 eyes, Group B included 105 patients and 135 eyes, and Group C included 25 patients and 30 eyes. No statistically significant differences were observed among the 3 groups in sex distribution or laterality. The baseline characteristics of the study population are summarized in Table 1.

**Table 1:** Baseline characteristics of children undergoing lacrimal duct probing for CNLDO.

Age group	Total cases, n	Eyes, n (%)	Male patients, n	Male eyes, n	Female patients, n	Female eyes, n
Group A (3–6 months)	174	229 (58.1%)	95	124	79	105
Group B (7–12 months)	105	135 (34.3%)	56	68	49	67
Group C (13–24 months)	25	30 (7.6%)	14	16	11	14
Total	304	394 (100.0%)	165	208	139	186

CNLDO = congenital nasolacrimal duct obstruction.

This study was approved by the Ethics Committee of Tongzhou Maternal and Child Health Hospital of Beijing. The requirement for additional study-specific informed consent was waived because of the retrospective nature of the study. The study was conducted in accordance with the principles of the Declaration of Helsinki. Written informed consent for lacrimal duct probing was obtained from the guardians of all patients.

### 2.2. Inclusion and exclusion criteria

The inclusion criteria were as follows: diagnosis of CNLDO based on persistent tearing since birth, recurrent conjunctival redness, increased mucopurulent discharge, and reflux of mucopurulent secretions after digital pressure over the lacrimal sac; diagnostic confirmation by lacrimal irrigation showing regurgitation of mucopurulent material or saline; age between 3 and 24 months at the time of probing; and complete clinical records.

The exclusion criteria were as follows: absence of both upper and lower lacrimal puncta; bony nasolacrimal duct anomalies; nasal malformations or other congenital nasal diseases; premature birth; very low birth weight; presence of other severe systemic disorders; or incomplete clinical or follow-up data.

### 2.3. Conservative management and criteria for probing

Before probing, conservative treatment, including lacrimal sac massage and topical antibiotics when clinically indicated, was recommended for patients with CNLDO. Lacrimal duct probing was performed in patients with persistent symptoms despite conservative management or when intervention was considered clinically appropriate based on symptom severity, recurrent mucopurulent discharge, parental preference, and the surgeon’s clinical judgment.

After probing, patients were not advised to continue nasolacrimal duct massage. Postoperative management consisted of topical antibiotic eye drops and scheduled follow-up assessments. Therefore, postoperative massage performance was not considered a factor influencing the need for repeat probing in this cohort.

### 2.4. Surgical procedure

All affected eyes were treated with topical antibiotic eye drops for 1 week before surgery. On the day of the procedure, 0.4% benoxinate hydrochloride was instilled into the conjunctival sac for topical surface anesthesia. No systemic sedation or general anesthesia was used. A 5 mL syringe filled with normal saline was prepared for lacrimal irrigation.

With the child in the supine position, the head was gently stabilized by an assistant. The surgeon exposed the inferior lacrimal punctum using the index finger and thumb, and the punctum was dilated using a lacrimal dilator. A No. 6 or No. 7 lacrimal probe was selected according to the size of the lacrimal punctum and canaliculus, as judged by the surgeon.

The probe was inserted gently through the inferior lacrimal punctum into the canaliculus and advanced along the anatomical course of the lacrimal drainage system. Lacrimal irrigation with normal saline was performed to confirm obstruction and assess patency. If obstruction was confirmed, the probe was advanced carefully through the nasolacrimal duct until a breakthrough sensation was felt, indicating passage through the obstructed segment. Successful passage was further confirmed by saline flow into the nasal cavity or by observation of a swallowing response.

The probe was then withdrawn slowly. A drop of antibiotic solution was administered postoperatively, and parents were instructed to instill topical antibiotic eye drops 4 times daily for 1 week. Routine nasolacrimal duct massage was not recommended after probing.

All procedures were performed by experienced ophthalmic surgeons. During the procedure, the child was monitored for respiratory distress, cyanosis, aspiration, bleeding, false passage, or other complications. If clinically significant respiratory or procedural complications occurred, the procedure would be stopped immediately and appropriate airway positioning, suction, or pediatric support would be provided. No cases of intraoperative apnea, cyanosis, or aspiration pneumonia were observed in this cohort.

### 2.5. Outcome assessment

The primary outcome was short-term clinical resolution after lacrimal duct probing. Short-term clinical resolution was defined as absence of epiphora and mucopurulent discharge, absence of reflux from the lacrimal sac after digital pressure, and patent lacrimal irrigation at postoperative follow-up. Outcome assessment was performed at 2 weeks after probing and confirmed at subsequent follow-up visits when available.

Repeat probing was considered for patients with persistent or recurrent symptoms and lack of patency on follow-up lacrimal irrigation. Because this was a retrospective study, no standardized symptom grading system, such as the Munk score, and no blinded outcome adjudication were used.

### 2.6. Statistical analysis

All data were analyzed using SPSS version 27.0 (IBM Corp., Armonk). Categorical variables were expressed as frequency and percentage.

Short-term clinical resolution rates and 95% confidence intervals (CIs) were calculated for the overall cohort and for each age group. Comparisons among groups were performed using the chi-square test or Fisher exact test, as appropriate. Pairwise comparisons were performed with Bonferroni correction. A 2-sided *P* value of <.05 was considered statistically significant.

Because this was a retrospective analysis of all eligible patients treated during the study period, no formal a priori sample size calculation was performed. The relatively small sample size in the 13 to 24 month group was considered when interpreting the results.

## 3. Results

A total of 304 pediatric patients with CNLDO, involving 394 affected eyes, underwent lacrimal duct probing during the study period. According to age at the time of probing, patients were categorized into 3 groups: Group A, 3 to 6 months, 229 eyes; Group B, 7 to 12 months, 135 eyes; and Group C, 13 to 24 months, 30 eyes.

Overall, 386 of the 394 treated eyes achieved short-term clinical resolution, corresponding to an overall success rate of 98.0% (95% CI: 96.0–99.1%). A total of 344 eyes achieved resolution after the first probing, yielding a first-procedure success rate of 87.3% (95% CI: 83.6–90.4%). Among the 50 eyes that required a second probing, 42 achieved resolution, corresponding to a second-procedure success rate of 84.0% (95% CI: 70.9–92.8%).

When analyzed by age group, the success rate after the first probing was 86.5% (198/229) in Group A, 90.4% (122/135) in Group B, and 80.0% (24/30) in Group C. Among eyes requiring a second probing, the success rate was 96.8% (30/31) in Group A, 76.9% (10/13) in Group B, and 33.3% (2/6) in Group C. These results are shown in Table 2.

**Table 2:** Short-term clinical resolution after first and second probing in each age group.

Age group	Total eyes, n (%)	Resolved after 1st probing, n	1st probing success rate	Eyes requiring 2nd probing, n	Resolved after 2nd probing, n	2nd probing success rate
Group A (3–6 months)	229 (58.1%)	198	86.5%	31	30	96.8% (30/31)
Group B (7–12 months)	135 (34.3%)	122	90.4%	13	10	76.9% (10/13)
Group C (13–24 months)	30 (7.6%)	24	80.0%	6	2	33.3% (2/6)
Total	394 (100.0%)	344	87.3%	50	42	84.0% (42/50)

The denominator for the second probing success rate is the number of eyes that underwent a second probing after persistent or recurrent symptoms following the first procedure.

The total short-term clinical resolution rate, including both first and second probings, was 99.6% (228/229; 95% CI: 97.6–100%) in Group A, 97.8% (132/135; 95% CI: 93.6–99.5%) in Group B, and 86.7% (26/30; 95% CI: 69.3–96.2%) in Group C. A significant difference was observed among the 3 groups (*P* < .001). Pairwise comparisons showed that the success rates in Groups A and B were significantly higher than that in Group C (*P* < .05), whereas no statistically significant difference was observed between Groups A and B (*P* > .05). Detailed comparisons are presented in Table 3.

**Table 3:** Comparison of total short-term clinical resolution rates by age group.

Age group	Total eyes, n	Resolved eyes, n (%)	95% CI	*P* value
Group A (3–6 months)	229	228 (99.6%)	97.6–100%	
Group B (7–12 months)	135	132 (97.8%)	93.6–99.5%	<.001
Group C (13–24 months)	30	26 (86.7%)	69.3–96.2%	

The overall comparison among the 3 age groups was statistically significant. Pairwise comparisons showed that Group C had a significantly lower success rate than Groups A and B (*P* < .05), whereas no significant difference was observed between Groups A and B (*P* > .05).

CI = confidence interval.

Sex-specific analysis showed no significant difference in treatment outcomes. The short-term clinical resolution rate was 98.1% (204/208) among male patients and 97.8% (182/186) among female patients, with no statistically significant difference between sexes (*P* > .999).

No cases of intraoperative apnea, cyanosis, or aspiration pneumonia were observed in this cohort.

These findings indicate that lacrimal duct probing was associated with high short-term clinical resolution rates in infants with CNLDO, particularly in those treated within the first 12 months of life. However, because this was a retrospective study, the observed association between age group and treatment outcome should not be interpreted as proof that age alone determined procedural success.

## 4. Discussion

CNLDO is a common disorder in infants and is most frequently related to incomplete canalization or persistence of a membranous obstruction at the distal nasolacrimal duct, particularly near the valve of Hasner. Less commonly, bony nasolacrimal duct stenosis or nasal abnormalities may contribute to obstruction.^[12,13]^ Persistent tear retention may lead to recurrent mucopurulent discharge, conjunctival irritation, and secondary infection, which can affect quality of life for both infants and caregivers.

Conservative management, including lacrimal sac massage and topical antibiotics when clinically indicated, remains the initial approach for many infants with CNLDO. This is consistent with previous reports showing that many cases resolve spontaneously during infancy.^[14]^ When symptoms persist despite conservative management, lacrimal duct probing is widely used as the initial surgical intervention and has been reported to be effective in many pediatric patients.^[15–18]^

The timing of probing remains clinically important. Current guidelines and previous studies generally support initial conservative treatment in early infancy, while recognizing probing as an appropriate option for persistent obstruction.^[9]^ Some studies have suggested that delayed probing may be associated with lower success rates, possibly because long-standing obstruction can lead to chronic inflammation, fibrosis, or more complex anatomical resistance.^[19–23]^ Other studieshave reported that probing performed after 6 or 9 months of age may still achieve favorable outcomes.^[24–27]^ Therefore, the decision regarding timing should consider the likelihood of spontaneous resolution, symptom severity, parental preference, and the potential risk of reduced success with prolonged obstruction.

In the present study, lacrimal duct probing was associated with a high short-term clinical resolution rate. The overall success rate was 98.0%, and the total success rates were 99.6% in infants aged 3 to 6 months, 97.8% in those aged 7 to 12 months, and 86.7% in those aged 13 to 24 months. No significant difference was observed between the 3 to 6 month and 7 to 12 month groups, whereas both groups had significantly higher success rates than the 13 to 24 month group. These findings suggest that probing within the first year of life can achieve favorable short-term outcomes in infants with persistent CNLDO.

Our findings are generally consistent with established guidance, including the clinical guideline by Sasaki et al,^[9]^ in that they support conservative management as the initial approach for many infants and probing as an effective intervention when symptoms persist. The practical contribution of our study is not to contradict existing guidelines, but to provide additional real-world evidence from a single-center cohort comparing outcomes across 3 age groups. In particular, our results suggest that probing during the 3 to 12 month window was associated with similarly high short-term success rates, whereas outcomes appeared less favorable after 12 months.

These findings may help guide clinical decision-making in daily practice. For infants with persistent tearing, mucopurulent discharge, or recurrent symptoms despite conservative treatment, probing can be considered within the first year of life rather than being delayed beyond 12 months. However, the decision should remain individualized. Factors such as symptom severity, response to lacrimal sac massage, frequency of infection, parental preference, and surgeon experience should be considered when selecting the timing of intervention.

This study has several limitations. First, because of the retrospective design, patients treated at different ages may have differed in disease severity, response to conservative management, parental preference, referral timing, and other unmeasured factors. Therefore, the association between age at probing and treatment outcome should not be interpreted as proof that age alone determined procedural success. Second, the outcome was assessed mainly according to short-term clinical resolution and lacrimal irrigation findings. The follow-up duration was insufficient to fully evaluate recurrence, late complications, or sustained long-term nasolacrimal duct patency. Third, the 13 to 24 month group included only 30 eyes, resulting in wider confidence intervals and limited statistical power. Fourth, no standardized symptom score, such as the Munk score, and no blinded outcome adjudication were used. Patency assessment by lacrimal irrigation may be operator dependent. Finally, because detailed adherence to preoperative massage was not consistently recorded, we could not assess whether the quality or duration of conservative management influenced the need for repeat probing.

Despite these limitations, this study adds practical evidence supporting timely probing for persistent CNLDO during infancy. Future prospective, multicenter studies with larger samples, longer follow-up, standardized symptom scoring, and adjustment for potential confounders are needed to better define the optimal timing of probing and to evaluate long-term outcomes.

## 5. Conclusion

Lacrimal duct probing was associated with a high short-term clinical resolution rate in infants with CNLDO, particularly when performed within the first 12 months of life. In this retrospective cohort, outcomes were less favorable in patients treated after 12 months compared with those treated at 3 to 12 months. These findings support consideration of timely probing for persistent CNLDO that does not respond to conservative management. However, because of the retrospective design, limited follow-up duration, and small number of patients older than 12 months, further prospective studies with longer follow-up and adjustment for potential confounders are needed to confirm the optimal timing of intervention.

## Author contributions

**Conceptualization:** Xiaolan Xie, Bidan Zhu.

**Data curation:** Xiaolan Xie, Shana Wang, Xi Qin, Jingjing Wang.

**Formal analysis:** Shana Wang, Jingjing Wang.

**Funding acquisition:** Bidan Zhu.

**Investigation:** Shana Wang, Xi Qin, Jingjing Wang.

**Methodology:** Xiaolan Xie, Shana Wang, Xi Qin.

**Supervision:** Bidan Zhu.

**Writing – original draft:** Xiaolan Xie.

**Writing – review & editing:** Bidan Zhu.
